# HOME HEALTH USE BY OLDER ADULTS WITH DEMENTIA: COMPARING MEDICARE ADVANTAGE AND TRADITIONAL MEDICARE

**DOI:** 10.1093/geroni/igae098.2156

**Published:** 2024-12-31

**Authors:** Jamie Smith, Jianhui (Frank) Xu, Teneil Brown, Julia Burgdorf, Daniel Polsky, Katherine Ornstein

**Affiliations:** Widener University, Chester, Pennsylvania, United States; Johns Hopkins University, Baltimore, Maryland, United States; Johns Hopkins University, Baltimore, Maryland, United States; VNS Health, New York, New York, United States; Johns Hopkins University, Baltimore, Maryland, United States; Johns Hopkins University, Baltimore, Maryland, United States

## Abstract

Skilled home health care (HHC) is a critical service for community-dwelling older adults with dementia, but the majority of evidence about its use and quality is based on Traditional Medicare (TM) beneficiaries. Medicare Advantage (MA) plans - which now enroll over half of Medicare beneficiaries - have flexibility in financing and managing HHC, potentially affecting access and utilization. Given the growth in MA, increasing dementia prevalence, and delivery shifts to community settings, understanding how HHC use differs between MA and TM among persons with dementia is needed. This study compared HHC utilization in a nationally representative sample of persons with dementia in MA and TM. We used Medicare enrollment files linked to FY 2018-2019 MA encounter and TM claims data to identify persons 65+ years with dementia and at least one HHC visit in 2019. Our study sample included 316,407 persons with dementia (66% TM vs 34% MA). MA enrollees were more likely to identify as non-Hispanic, Black (13% vs. 10%), or Hispanic (12% vs. 6%) than TM. Greater proportion of TM beneficiaries were dual eligible (37% vs. 32%). The groups were similar in age, and 34% were male. Overall, MA enrollees were less likely to use HHC services than TM beneficiaries (26.2% vs 29.8%). This presentation will discuss variation in home health visit type/intensity and findings stratified by MA plan type, carriers, and geographic distribution. We will share novel approaches and best practices for measuring HHC utilization among people with dementia using newly available MA encounter data.

